# Synthetic lipoprotein as nano-material vehicle in the targeted drug delivery

**DOI:** 10.1080/10717544.2017.1384518

**Published:** 2017-10-25

**Authors:** Xueqin Zhang, Gangliang Huang

**Affiliations:** Active Carbohydrate Research Center, College of Chemistry, Chongqing Normal University, Chongqing, PR China

**Keywords:** High-density lipoprotein, low-density lipoprotein, recombinant lipoprotein, nano-scaled vehicles, targeted drug delivery

## Abstract

High-density lipoprotein (HDL) and low-density lipoprotein (LDL), as human endogenous lipoprotein particles, have low toxicity, high selectivity, and good safety. They can avoid the recognition and clearance of human reticuloendothelial system. These synthetic lipoproteins (sLPs) have been attracted extensive attention as the nanovectors for tumor-targeted drug and gene delivery. Herein, recent advances in the field of anticancer based on these two lipid proteins and recombinant lipoproteins (rLPs) as target delivery vectors were analyzed and discussed.

## 1. Introduction

The application of nanotechnology in drug delivery is a new hotspot in the field of drug delivery in recent years (Singh, 2013), and the bionic vehicle attracts more people’s attention because of its good security. Lipoprotein is the natural vehicle of lipid transport in human body. Its main components are lipoproteins, phospholipids, cholesterol esters, free cholesterol, and protein. Lipoproteins in the body mainly have chylomicrons, very low density lipoprotein (VLDL), low density lipoprotein (LDL), and high density lipoprotein (HDL). The biomimetic nano-delivery system of drug based on lipoprotein has attracted wide interest because of its excellent *in vivo* compatibility and natural targeting. Counsell et al. (2009) also began to work on the study of lipoprotein vehicles, such as the use of LDL complexes in the transport of X-ray contrast agents. In recent years, the research of nano-delivery system based on synthetic lipoprotein (sLP) and recombinant lipoprotein (rLP) has been widely developed. Herein, the advances in drug delivery systems based sLPs were analyzed and discussed.

## 2. Characteristics of sLP as nano-material vehicle of drug

### 2.1. The biocompatibility is good and the structure is satisfactory

Compared with other nanoparticles (NPs), the sLP has the significant advantage. First, the sLP NPs have good biocompatibility. They not only can avoid to be identificated in the reticuloendothelial system for rapid clearance, but also can be completely degraded *in vivo* (Ng et al., 2011). Second, the sLP was constructed by the hydrophilic surface and hydrophobic core, which is conducive to the transport of hydrophobic substances. sLP in the blood can cycle for a long time, and its NP size is appropriate (less than 30 nm), which is not only small enough to penetrate the tumor fiber space, but also can avoid to be rapidly eliminated by kidney due to too small particle size. Therefore, it is a good drug nano-material vehicle, who can maintain the curative effect for a long time (Xu et al., 2010; Nam et al., 2016).

### 2.2. The mechanism cellular trafficking of sLPs

The apolipoprotein on the surface of LDL and HDL can be specific to identify the corresponding receptor of target cell, which can give a targeting delivery for drug to the specific type of cell, and thus it has a good targeting. Lipoprotein receptor can be divided into two categories according to the function: The first category is the receptor of endocytosis, such as low-density lipoprotein receptor (LDLR), LDLR-related protein, and scavenger receptor type A (SRA); The second category is the receptor that regulates the lipid exchange of cell membrane, including scavenger receptor class B type I (SR-BI) (encoded by the *SCARB*1 gene), scavenger receptor class B type II (SR-BII) (encoded by the *SCARB2* gene), and CD36 (Brodeur et al., 2008). The researchers found that these receptors of LDL and HDL widely existed in the surfaces of various types of tumor cells (Berkel et al., 1999; Van Berkel et al., 2000). For example, it was showed that the delivery of lipophilic drugs by LDL might provide distinct advantages over the use of synthetic vehicles (Samadi-Baboli et al., 1990). In the case of scavenger receptor class B type 1 (SR-B1), it indicated that the expression level of SR-B1 in the LNCaP tumor animal model was two times higher than that of the normal animal model (Chen et al., 2006). At the same time, there is evidence that SR-BI is a potential biomarker for nasopharyngeal carcinoma (Oluwadara & Chiappelli, 2009). High level expression of SR-B1 was also found in prostate, breast, colorectal, and ovarian cancers. Therefore, the researchers can use the lipoprotein receptors expressed in tumor cells as targets for diagnosis or treatment of tumors. Remarkably, these lipoprotein receptors are also present on the cell surfaces of non-neoplastic diseases (Nanjee & Miller, 1989). So, it should also pay attention to the expression of lipoprotein receptor in normal tissue and avoid causing the cell toxicity from outside the target range in drug delivery with LDL or HDL as the vehicle.

### 2.3. The stability of sLPs

siRNAs were bonded to the lysine polypeptides to neutralize the charge. Then they were wrapped up in recombinant HDL (rHDL) kernel, and 4 mg siRNAs/mL (entrapment rate >90%) of the loading capacity with relative stability was obtained (Hinze, 2013).

Versluis et al. (1999) incorporated a lipophilic derivative of daunorubicin (LAD) into the apolipoprotein E (ApoE)-exposing liposomes. Compared with free LAD, the circulation half-life of the liposome-associated prodrug was substantially prolonged and tissue disposition was reduced. In rat-model, the liver uptake of the prodrug and vehicle was increased about 5-fold. In LDL receptor-deficient mice-model, compared with wild-type mice, the circulation time of both the prodrug and the vehicle increased about 2-fold. Therefore, it proved that LAD-loaded ApoE liposomes might be a stable drug-vehicle complex, which was well suited for the selective drug delivery mediated by LDL receptor for tumor therapy.

## 3. Preparation of sLP as drug nano-material vehicle

Masquelier et al. (1989) reported the preparation of recombinant complexes using LDL and hydrophobic compounds. McConathy et al. (2008) prepared the rHDL particles carrying paclitaxel (PTX) (rHDL/PTX) with higher PTX content. The application of recombinant LDL (rLDL) is limited by the large size of ApoB-100 and its complexity of preparation. Therefore, the researchers have found that ApoB-100 mimic peptide can be used to replace ApoB-100 for the synthesis of LDL.

### 3.1. The natural lipoprotein and sLP

In the early stage, the natural lipoprotein was used as nano-material vehicle of drug, and it could be isolated directly from the donor body. Drug, photosensitizer (Jori & Reddi, 1993), nucleoside (Hammel et al., 2003), and fluorescent imaging agent (Li et al., 2005) loaded in the natural lipoproteins NPs have effects *in vitro* and *in vivo*. However, their application is still restricted by some factors, including the separation step, storage process, drug loading (Shaw & Shaw, 1991), safety, and other problems. Therefore, the development of natural lipoprotein NPs gradually shifts to the sLP. Compared with natural lipoprotein, lipoprotein has the unique advantages. In the first place, all compounds that constitute the sLPs are known, so, they can be characterized for their respective structures. Furthermore, a class of compounds in sLP can be adjusted separately, such as stoichiometry ratio of lipid/protein, kind of lipid and apolipoprotein, which can regulate the physicochemical properties of lipoprotein NPs, such as size, zeta potential, core, and surface load. Finally, these sLPs also possess the known functions of the native lipoproteins, for example, natural targeting, activation of enzyme channels, increased cellular uptake, lipid transfer, etc.

### 3.2. The synthetic LDL (sLDL) and the synthetic HDL (sHDL)

Nikanjam et al. (2007) constructed the nano-LDL (nLDL) containing PTX oleate (nLDL-PO). It indicated that the nLDL-PO cell survival in glioblastoma multiforme (GBM) cell lines was the time, concentration, and cell line dependent. The PTX was delivered via the LDL receptor. It was suggested that the synthetic nLDLs could incorporate lipophilic drugs to kill GBM cells. At the same time, people are trying to study how the sLDL NPs were used for the delivery of drug to targeting tumor cell. Moreover, researchers are considering to utilize the sHDL NPs. Earlier studies showed that fluorouracil, iododeoxyuridine, doxorubicin, and vindesine could be successfully encapsulated into sHDL, which had the lower IC_50_, and cellular uptake ability was significantly increased with the increase of surface lipoprotein receptor expression level (Lacko & Mcconathy, 2009). In addition, the regulation of HDL receptor (SR-BI) can cause the inactivation of lysosomal pathway, and thus enhance the interference of RNA. So, the sHDL is more favorable for the effective delivery of siRNAs (Lin et al., 2012; Rui et al., 2013). A large number of studies have shown that the sHDL NPs can be used as good vehicle of anti-tumor drugs, and also successfully applied to other diseases. Shin et al. (2012) developed a pH-responsive high-density lipoprotein (HDL)-like NP, which could selectively releases PTX. As a result of the pH responsiveness, the anticancer effect of PTX NPs was much more potent than free PTX or HDL-like NP containing phospholipids, phosphatidylcholine, and apolipoprotein A-I (apo A-I), as well as PTX. It was speculated that the pH responsiveness of PTX NPs enabled efficient endosomal escape of PTX before lysosomal broke down.

### 3.3. The sLP and analog lipoprotein

Although sLP can be used as a suitable drug nano-material vehicle, but the apolipoproteins (ApoB-100, ApoA-I, and ApoE) are not easy to extract, and are difficult to obtain, which restricts the synthesis of artificial lipoproteins. So, this brings new problems. Based on the high similarity of recombinant apolipoprotein and natural apolipoprotein, it can solve this limitation to use peptides with similar properties and functions of apolipoprotein. People found that ApoB-100 was cloned to express vector, resulting in a peptide containing 560 amino acids, it could be used to simulate the ApoB-100 polypeptide for the synthesis of LDL. Similarly, the application of analog ApoA-I peptides (produced by bacteria, yeast, and plant systems) solves the problem of difficult separation of natural ApoA-I, which greatly increases the use of sHDL in drug delivery systems. Therefore, it further promotes the research and development of sLP as drug nano-material vehicle with the simulated lipoprotein synthesized with mimic peptide (Navab et al., 2015).

## 4. Utilizations of sLP as drug nano-material vehicle

### 4.1. rLPs

rLPs are the most widely used in sLPs. They are formed by the combination of well separated apolipoproteins (recombinant or naturally derived) and a variety of natural or synthetic lipids. rLP mainly includes rLDL and rHDL, its particle size is less than 30 nm of endogenous nano-material vehicle with a large capacity of hydrophobic core, which is conducive to provide a stable vehicle space. In addition, the two can be used for the active target therapy of tumor cells through the receptor-mediated pathway (Rensen et al., 2001).

#### 4.1.1. rLDL

It is noted that the rLDL NPs are more effective in promoting cell selective uptake of drug than the native LDL. Today, the recombinant ApoE has been used to synthesize LDL NPs, making it a bionic vector for targeted delivery of drugs.

Earlier studies showed that the rLDL could encapsulate fluorescent probes, chemotherapy drugs (Hayavi & Halbert, 2005), and siRNAs (Kim et al., 2008). Recently researchers have begun to attach importance to the application of rLDL as a fluorescent agent or imaging agent in the transmission system. For example, Nikanjam et al. (2007) developed a synthetic nLDL particle, which could be used as a drug delivery vehicle for targeting GBM tumors via the LDLR. It indicated that the uptake of nLDL was time-dependent and concentration-dependent manner. Zhou et al. (2010) investigated the uptake extent of sLDL by leukemic cell lines and chronic myeloid leukemia (CML) patient stem/progenitor cells. Compared with Bcr-Abl negative, it showed an increased and preferential uptake of sLDL by Bcr-Abl positive cell lines. In addition, CML CD34^+^ and primitive CD34^+^38^lo/−^ cells accumulated significantly higher levels of sLDL in comparison to non-CML CD34^+^ cells. Therefore, the drug-loading sLDL NPs could potentially enhance intracellular drug concentrations in primitive CML cells. So, this aided their eradication. Su et al. (2016) designed a synthetic LDL (sLDL) for encapsulating PTX-alpha linolenic acid (PALA), which was used for tumor therapy. Compared with PALA-loaded microemulsion (PALA-ME), it was showed by *in vitro* studies that PALA-loaded sLDL (PALA-sLDL) exhibited the enhanced cellular uptake capacity and better cytotoxicity to LDLR over-expressed U87 MG cells. In addition, it indicated that PALA-sLDL had higher tumor accumulation than PALA-ME, and its tumor inhibition efficiency was higher than that of Taxol^®^ and PALA-ME, respectively. In addition, PALA-sLDL had the lower toxicity. So, it proved that sLDL could be used as a valuable vehicle to selectively deliver anticancer drugs for the tumor therapy. In summary, these researchers have successfully wrapped up all sorts of chemical drugs, biological drugs, and contrast agents into reconstituted LDL, which retained the similar to *in vitro* and *in vivo* biological function of native LDL, and expanded the types of NPs as drug vehicle targeting cells.

#### 4.1.2. rHDL

rHDL is produced by the self-assembly of natural or recombinant ApoA-I or ApoE and phospholipid/cholesterol ester/cholesterol emulsion, and is nanodisc-like or spherical NPs (Kanwar et al., 2012). The drug loaded on the NPs, whether is encapsulated in the core or bonded to the surface, can be added directly into the emulsion, which forms particles later or in advance. The obtained NPs thus retain the physical and chemical properties, and biological functions of natural HDL. rHDL NPs used as general drug delivery tools can be equipped with various types of chemotherapy drugs, siRNAs, photosensitizers, and imaging agents. Compared with other NPs, rHDL is an excellent vehicle because it has smaller particle size (about 10 nm), natural targeting ability (SR-BI receptor), unique transmission path, simple chemical interface, and ability to wrap up the hydrophobic molecules.

A typical model of rHDL is made of egg yolk lecithin, cholesterol, phospholipid, and recombinant ApoA-I. The performance and size (11.4 nm) of NPs obtained by this way are similar to that of natural HDL. Yuan et al. (2016) developed a novel 10-hydroxycamptothecin (HCPT) delivery system of sHDL NP to treat colon carcinoma. HDL was recognized by SR-BI, which was over-expressed in colon carcinomas. It was showed that the IC_50_ of HCPT-sHDL was about 3-fold lower than that of free HCPT by the cytotoxicity studies with HT29 colon carcinoma cells. It also indicated that the area under the serum concentration-time curve (AUC_0–t_) and Cmax of HCPT-HDL was 2.7 or 6.5-fold higher than that of free HCPT, respectively. Therefore, sHDL-based formulations of hydrophobic drugs would be useful to treat SR-BI-positive tumors. The rHDL surface was engaged with folic acid, which was used to verify whether the modified of rHDL was targeted to folate receptor or not (Corbin, 2013). The results showed that changing the rHDL NP targeting route was completely feasible to target to other receptors except the SR-BI. At the same time, it was confirmed that the rHDL drug nano-material vehicle enhanced the efficacy of PTX in the treatment of ovarian cancer, prostate cancer and breast cancer *in vitro*, and reduced the toxicity of PTX on normal cells in C57/BL6 mice. In addition, the potential of rHDL in terms of delivery of siRNAs has also been fully developed. At present, synthetic HDL has been extended to the application for a variety of drugs, such as the vitamin A for treatment of nerve cell tumors, valrubicin for strengthening the treatment of prostate cancer, ovarian cancer, etc. (Shah et al., 2016).

Amyloid beta (Aβ) and its aggregation forms in the brain are the key targets to treat Alzheimer’s disease (AD). So, developing the nano-material vehicle, which can permeate blood-brain barrier and target Aβ, is very important to intervent AD. Song et al. (2016) constructed the ApoE-reconstituted high density lipoprotein nano-material carrier (ANC) to achieve the above-mentioned goals. α-Mangostin (α-M) can inhibit to form Aβ oligomers and fibrils, it can also accelerate to degrade Aβ cell. So, α-M was used as the model drug. Compared with the control liposome, ANC had approximately 54-fold higher cellular uptake in brain endothelial cell line *in vitro*, which was in an ApoE-dependent manner. In addition, it had much higher brain delivery efficiency *in vivo*. It indicated that ANC-α-M had the most efficient efficacy to decrease amyloid deposition, attenuate microgliosis, and rescue memory defect in SAMP8 mice, which was an AD mouse model. Therefore, it proved that the ApoE-based biomimetic nano-material vehicle could offer a promising platform for brain drug delivery to treat AD.

The rHDL nanodiscs have been widely used in drug delivery systems. A rHDL nanodisc model was proposed for the ApoA-I receptor, which could be used for the inclusion and delivery of curcumin (Khumsupan et al., 2011). Curcumin is an active component of traditional Chinese medicine, but it has poor oral absorption and low bioavailability. The nanodisc was beneficial to enhance the water solubility of curcumin and enhanced its therapeutic effect (Simonsen, 2016). It indicated that the curcumin nanodisc could bring about 70% apoptosis in the mantle cell lymphoma (MCL), which was significantly increased than that of free curcumin (20%) and blank nanodisc (<10%) (Crosby et al., 2015). In addition, if ApoE was replaced by ApoA-I, the effect of curcumin on GBM cells could be significantly enhanced. The researchers found that the modification of lipid components on the rHDL nanodiscs could obtain some unique properties. A new rHDL disc model (10 ∼ 30 nm) was presented, which used porphyrin-lipid as only lipid compound for the connection of ApoA-I, ApoE3, or protein (Garai et al., 2014). When the obtained porphyrin nanodisc maintained a complete particle state, the photosensitivity of porphyrin would highly quench, but when the nanostructures were destroyed, the fluorescence and singlet oxidation could be quickly restored. Therefore, porphyrin nanodisc could not only be used as a fluorescent imaging probe, but also could be used as a photosensitive agent in optical therapy.

In addition to the above examples, rHDL is also used for the delivery of other drugs, including the delivery of fungicides, antimicrobial agents, viruses, and antiviral substances in a variety of cell and animal models. rHDL also has good application prospects in the treatment of AD. The rHDL/PTX NPs had superior cytotoxicity to kill several cancer cell lines, and the IC_50_ was 5–20 times lower than that of free PTX. It indicated that the uptake of PTX was facilitated by the scavenger receptor type B-1 (SR-B1) when drug-loaded synthetic/reconstituted high density lipoprotein (rHDL) particles were incubated with cells, which could express the SR-B1 receptor (Vassiliou & Mcpherson, 2004).

### 4.2. Simulated lipoprotein NPs prepared with simulated peptide

Although synthetic rLPs increase the feasibility of lipoproteins as drug vehicles, but their applications are still subject to limitations because the synthesis of apolipoproteins (ApoB-100 and ApoA-I) needs longer time and has more cumbersome working procedure, for example, removing modified groups in recombinant protein and purifying secreted proteins need additional steps. Therefore, researchers have used peptide, which simulate the functions and properties of apolipoprotein, to develop other types of sLPs. The biggest advantage of simulating sLP is to overcome the deficiency of apolipoprotein, such as purity, quantity, long processing time, safety, and other problems. Therefore, the simulated lipoprotein synthesized with simulated peptide can simplify the production process, and accelerate the application of lipoprotein-based drug delivery system in clinical research.

#### 4.2.1. Simulated LDL NPs

The oleic acid ester of PTX was introduced into the core of nLDL (nLDL-PO) (Emami et al., 2012). *In vitro* GBM cell survival experiment showed that nLDL-PO inhibited the proliferation of tumor cells, and the inhibition rate was dependent on time, concentration and cell type. At the same time, if suramin was used, the apoptosis rate of nLDL-PO on the tumor cells would reduce, which was also confirmed that the NPs were transferred to the focal region by LDLR. Although the LDL drug delivery system has a broad prospect, its physical and chemical properties need to be further studied. In particular, LDL particles are not conducive to long-term storage, yet these shortcomings have to be explored and improved by researchers.

#### 4.2.2. Simulated HDL NPs

Although the recombinant apolipoprotein ApoA-I has been obtained from the bacterial expression system, many problems concerning the purity and quantity are also produced. Samples from the human body have the risk of being contaminated by pathogens, which also affects the application security of ApoA-I. Therefore, it is necessary to take complex and rigorous experimental procedures to ensure that the isolated ApoA-I is not contaminated by pathogens. In order to simplify the experimental procedure, people are conceiving to use synthetic peptide analogs based on ApoA-I amphipathic helical structure, although they cannot be directly copied the specific amino acid sequences of ApoA-I, their physicochemical properties are similar to ApoA-I and other apolipoprotein family. For example, 4 F polypeptide with 18 amino acid residues, is similar to the structure of ApoA-I, and has anti-inflammatory, anti-oxidative, anti-atherosclerosis, and anti-tumor effect.

Kim et al. (2007) established a potential apo A-I delivery system for nucleic acids to the liver, which was demonstrated by real-time *in vivo* imaging. The synthetic siRNAs, which could kill hepatitis B virus (HBV), were formulated into a complex, namely apo A-I and 1,2-dioleoyl-3-trimethylammonium-propane (DOTAP)/cholesterol (DTC-Apo). Then they were injected intravenously (IV) into a mouse model to carry replicating HBV. It was showed that administration of these NPs could reduce viral protein expression, which was caused by receptor-mediated endocytosis. Therefore, the interesting approach to siRNA delivery created a foundation to develop a new class of therapeutics for killing hepatitis viruses or hepatocyte genes, which were related to tumor growth. An Apo A-I mimetic peptide was used to develop the synthetic HDL simulation NPs. This was known as synthetic peptide-HDL-mimicking peptide-phospholipid scaffold (HPPS) NPs, which were formed by the self-assembly with ApoA-I mimic peptides, phospholipids and cholesterol esters (Lin et al., 2012). Like HDL, when there was no cholesterol ester and other hydrophobic vehicle, HPPS was nanodisc. But when the drug was included into HPPS, HPPS was spherical NPs with particle size of 10 ∼ 15 nm. The phospholipid or polypeptide was labeled with the hydrophobic dye DiR-BOA. It indicated that the fluorescence intensity of DiR-BOA was 55 times higher in SR-BI positive cells than that in SR-BI negative cells by confocal microscopy. If the natural HDL was added, 98% fluorescence intensity was inhibited. The *in vivo* studies were carried out to mice by subcutaneous injection with KB (SR-BI positive) and HT1080 (negative) tumor cells. It showed that HPPS was a priority in SR-BI positive tumor cells to accumulate, the fluorescence intensity in KB was 3.8 times higher than that in HT1080, and the accumulation was obvious after the injection for 72 h. In addition, the researchers found that DiR-BOA signal and lysosomal tracer were not for colocalization and subcellular fractionation studies, which confirmed that the phospholipid and polypeptide retained on the cell surface, and DiR-BOA enrichment site was in the cytoplasm rather than in the lysosomes. These suggested that the structural properties of HPPS and transport mechanism of targeted SR-BI were similar to the natural HDL.

In recent years, scientists have studied the transport mechanism of HPPS, verified the ability of HPPS to simulate HDL, and enhanced the understanding of HDL transmission route. In these studies, compounds on HPPS were fluorescently labeled, the scientists found that HPPS could specifically recognize and bind SR-BI receptor. After binding mediated with SR-BI, the fluorescent marker carried with HPPS was directly transported to the cytoplasmic matrix, rather than the whole HPPS internalization. Based on the HDL simulation ability and good stability, HPPS has been used as an effective vehicle of drug delivery, it can transport chemotherapy drugs, siRNAs and imaging contrast agents to tumor sites and enter tumor cells (Lin et al., 2014, 2016).

In addition to oncology, synthetic analog HDL with analog peptide also plays an important role in the treatment of AD. Studies have indicated that analog peptide R4F of oral ApoA-I can bind to amyloid β-protein (Aβ) in the brain, and form a polymer to reduce the accumulation of amyloid protein, thus improve the cognitive ability. Therefore, the R4F is used for the synthesis of HDLs, which can improve the biological utilization of polypeptides in treatment of diseases of the central nervous system (Yang et al., 2012).

It was revealed that the micelle mimics had the structural features of serum lipoproteins. Serum lipoproteins could solubilize many hydrophobic drugs, and they could be used as a biological transport system besides amphotericin B. So, the synthetic micelle mimics might achieve the same aim like lipoproteins. In addition, and the mimics were easy for structural modification, safe, and stabile (Lavasanifar et al., 2000).

## 5. Conclusion

Since lipoproteins were used in the diagnosis and as vehicles for drugs, the research about lipoprotein-based NP formulation has been more than 30 years. The use of sLPs as chemicals and gene vectors has been increasing in the treatment of cancer and cardiovascular diseases. sLP can be used for the transport of various substances and reagents, including chemotherapeutic drugs, antiviral agents, antibacterial agents, siRNAs, and imaging agents. Lipoprotein-based NPs have shown a series of advantages in the study, but there are still many problems to be solved. The difficulty in obtaining lipoproteins and apolipoproteins is one of the major reasons for their limited research and application. The synthesis of mimetic peptides with the function of lipoprotein receptor recognition has made great progress, which provides a new direction for further research and application of lipoprotein-based delivery system. The construction of a new type of apolipoprotein based on lipoprotein has also provided a new idea for the design of this drug delivery system (Shaw & Shaw, 1991; Huynh et al., 2009). The sLP-based NPs will be a promising platform for the diagnosis and the delivery of therapeutic drugs.
